# HLA-DPA1 gene is a potential predictor with prognostic values in multiple myeloma

**DOI:** 10.1186/s12885-020-07393-0

**Published:** 2020-09-24

**Authors:** Jie Yang, Fei Wang, Baoan Chen

**Affiliations:** Department of Hematology and Oncology, Zhongda Hospital, School of Medicine, Southeast University, No. 87, Dingjiaqiao, Gulou District, Nanjing, 210009 Jiangsu China

**Keywords:** Multiple myeloma, Hypoxia, Prognosis, Bioinformatics analysis

## Abstract

**Background:**

Multiple myeloma (MM) is an incurable hematological tumor, which is closely related to hypoxic bone marrow microenvironment. However, the underlying mechanisms are still far from fully understood. We took integrated bioinformatics analysis with expression profile GSE110113 downloaded from National Center for Biotechnology Information-Gene Expression Omnibus (NCBI-GEO) database, and screened out major histocompatibility complex, class II, DP alpha 1 (HLA-DPA1) as a hub gene related to hypoxia in MM.

**Methods:**

Differentially expressed genes (DEGs) were filtrated with R package “limma”. Gene Ontology (GO) enrichment and Kyoto Encyclopedia of Genes and Genomes (KEGG) pathway were performed using “clusterProfiler” package in R. Then, protein-protein interaction (PPI) network was established. Hub genes were screened out according to Maximal Clique Centrality (MCC). PrognoScan evaluated all the significant hub genes for survival analysis. ScanGEO was used for visualization of gene expression in different clinical studies. *P* and Cox *p* value < 0.05 was considered to be statistical significance.

**Results:**

HLA-DPA1 was finally picked out as a hub gene in MM related to hypoxia. MM patients with down-regulated expression of HLA-DPA1 has statistically significantly shorter disease specific survival (DSS) (COX *p* = 0.005411). Based on the clinical data of GSE47552 dataset, HLA-DPA1 expression showed significantly lower in MM patients than that in healthy donors (HDs) (*p* = 0.017).

**Conclusion:**

We identified HLA-DPA1 as a hub gene in MM related to hypoxia. HLA-DPA1 down-regulated expression was associated with MM patients’ poor outcome. Further functional and mechanistic studies are need to investigate HLA-DPA1 as potential therapeutic target.

## Background

Multiple myeloma (MM) is a hematological malignancy which is characterized by aberrant plasma cells infiltration in the bone marrow and complex heterogeneous cytogenetic abnormalities [1]. Accumulation of abnormal plasma cells replaces normal hematopoietic cells and leads to “CRAB” - hypercalcemia, renal failure, anemia, and bone lesions, even fetal outcome eventually [2]. With the deepening of basic and clinicalresearches, novel drugs mainly including proteasome inhibitors and immunomodulatory drugs have improved patients’ outcome to some extend [3, 4]. Besides, high-dose chemotherapy and tandem autologous stem cell transplant (ASCT), together with supportive care have significantly prolonged patients’ progression-free survival (PFS) and overall survival (OS) [5]. However, MM remains an uncurable disease as underlying molecular mechanisms of pathogenesis and progression are still largely unclear. Quite a few patients cannot get diagnosis and proper treatment in time. Therefore, identifying key mechanisms regulating MM is critically important for early diagnosis and targeted therapy.

With the advances of high-throughput platforms and microarray, more and more molecular heterogeneity on MM has been recognized [6, 7]. Hypoxia plays an important role in occurrence and development of MM [8, 9] and more related pathogenesis is still urgent needs to be explore for better diagnosis and treatment. In order to find potential biomarker of MM related to hypoxia, we analyzed the differentially expressed genes (DEGs) functions and pathways between normoxic and hypoxia-resistant (HR) MM cell lines contained in GSE110113 dataset. Major histocompatibility complex, class II, DP alpha 1 (HLA-DPA1) was finally screened out as a hub gene associated with poor outcome of MM related to hypoxia. In addition, survival analyses and gene expression level were visualized with online clinical data, and the results validated higher HLA-DPA1expression level of MM patients was associated with poor clinical outcome. The findings in this study provide new insights on HLA-DPA1 as a potential biomarker for MM and more research needs to be performed.

## Methods

### Data source and DEGs identification

Gene expression profile GSE110113 was downloaded from National Center for Biotechnology Information-Gene Expression Omnibus (NCBI-GEO) database (https://www.ncbi.nlm.nih.gov/geo/) [10]. The array data of GSE110113 were generated on GPL6244 platform (HuGene-1_0-st Affymetrix Human Gene 1.0 ST Array). There are four parental cells (RPMI8226, KMS-11, U266, IM-9) and four HR cells that derived from above parental cells. Two group cells were cultured under normoxic condition (20% O_2_) and hypoxic condition (1% O_2_) for 24 h, respectively.

R package “limma” was used to identify DEGs between normoxic and HR cells groups [11]. The screen criteria were adjusted *p* value < 0.05 and [log2FoldChange (log2FC)] > 1. All genes were visualized by volcanic maps and top 50 dramatically altered genes were selected to draw a heatmap by R package “ggplot2” [12].

### GO and KEGG analysis

Gene Ontology (GO) enrichment analysis and Kyoto Encyclopedia of Genes and Genomes (KEGG) pathway analysis were conducted by using R package “clusterProfiler” [13] which is for functional classification and gene clusters enrichment. GO enrichment includes biological process (BP), molecular function (MF), and cellular component (CC) three subontologies. Analysis results were displayed with “GOplot” package of R [14]. In addition, relationship between pathways was further analyzed with the ClueGO plug-ins of Cytoscape software 3.7.2 [15]. A *p* value less than 0.05 was considered statistically significant.

### PPI network analysis

To clarify the relationships among proteins encoded by selected enrichment genes, a protein-protein interaction (PPI) network was established using the STRING database (https://string-db.org) [16]. Cytoscape software 3.7.2 was used to visualize the genes with minimum interaction score more than 0.4 [15]. Then, we utilized cytoHubba plug-ins to recognize interaction degree of hub-gene clustering according to the Maximal Clique Centrality (MCC) methods. Wayne diagram produced by online tool Bioinformatics & Evolutionary Genomics (http://bioinformatics.psb.ugent.be/webtools/Venn/) was used to show the overlapped genes.

### Survival analysis

To assess the prognostic value of selected genes in MM patients, survival analysis was performed with the PrognoScan database (http://dna00.bio.kyutech.ac.jp/PrognoScan/) [17]. PrognoScan explores the relationship between gene expression and prognosis of patients, across all the public available microarray datasets provided. The results are displayed with hazard ratio (HR) and Cox *p* value from a Log-rank test. Cox *p* value < 0.05 was considered statistically significant. Based on GSE2658 dataset (*n* = 559) provided by Zhan [18], relationship between gene expression and corresponding disease specific survival (DSS) were researched. Besides, according to online ScanGEO database (http://scangeo.dartmouth.edu/ScanGEO/) [19], we chose *p* value < 0.05 as significance criterion and screened out GSE47552 [20] and GSE2113 [21] datasets which involved HLA-DPA1 expression level compared to different degree of disease progression and healthy donors (HDs). Gene expression level in clinical patients was explored with the two datasets.

## Results

### Identification of DEGs

**Fig. 1:**
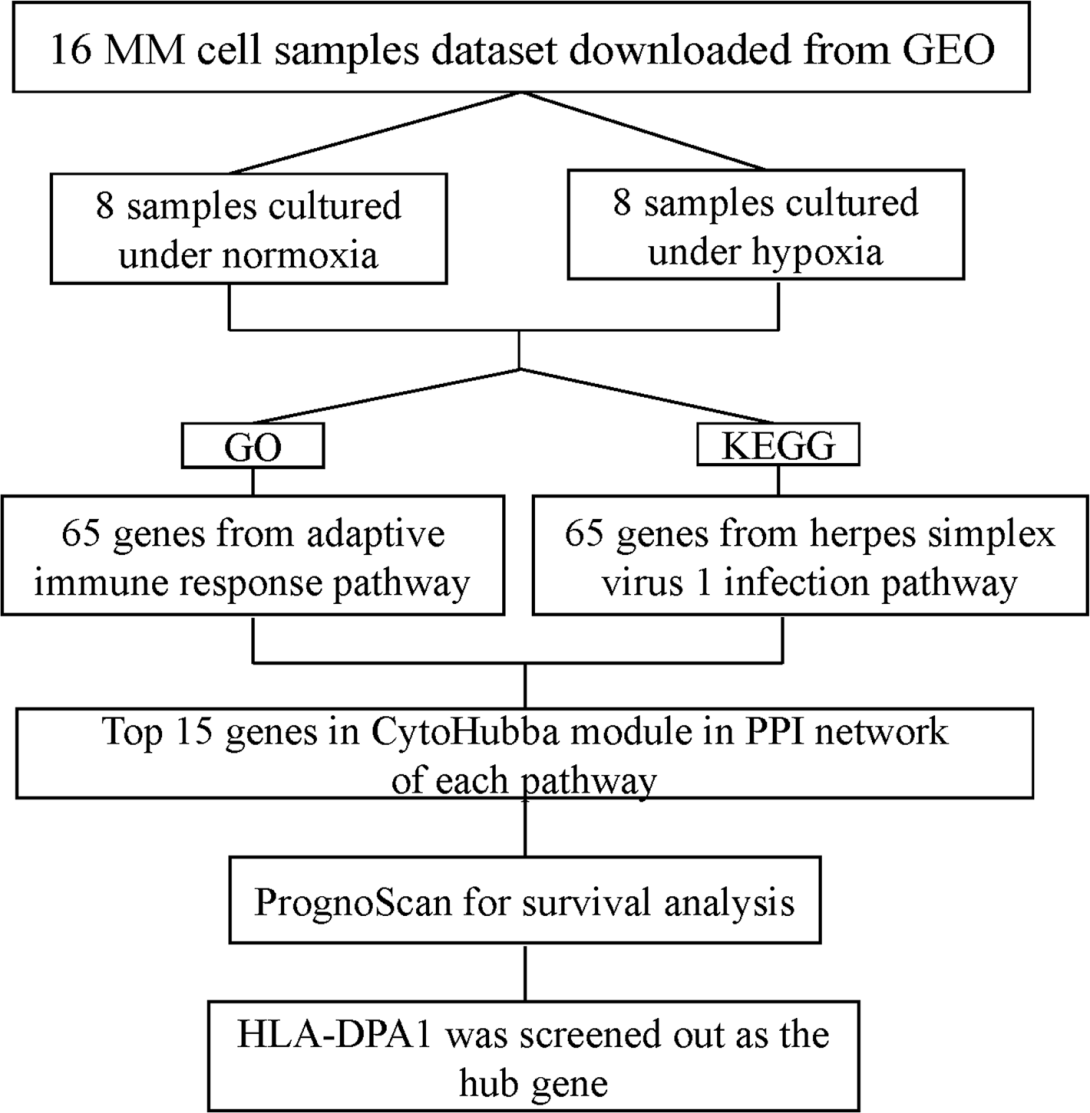
A schematic view of the procedure of the study with GSE110113

**Fig. 2:**
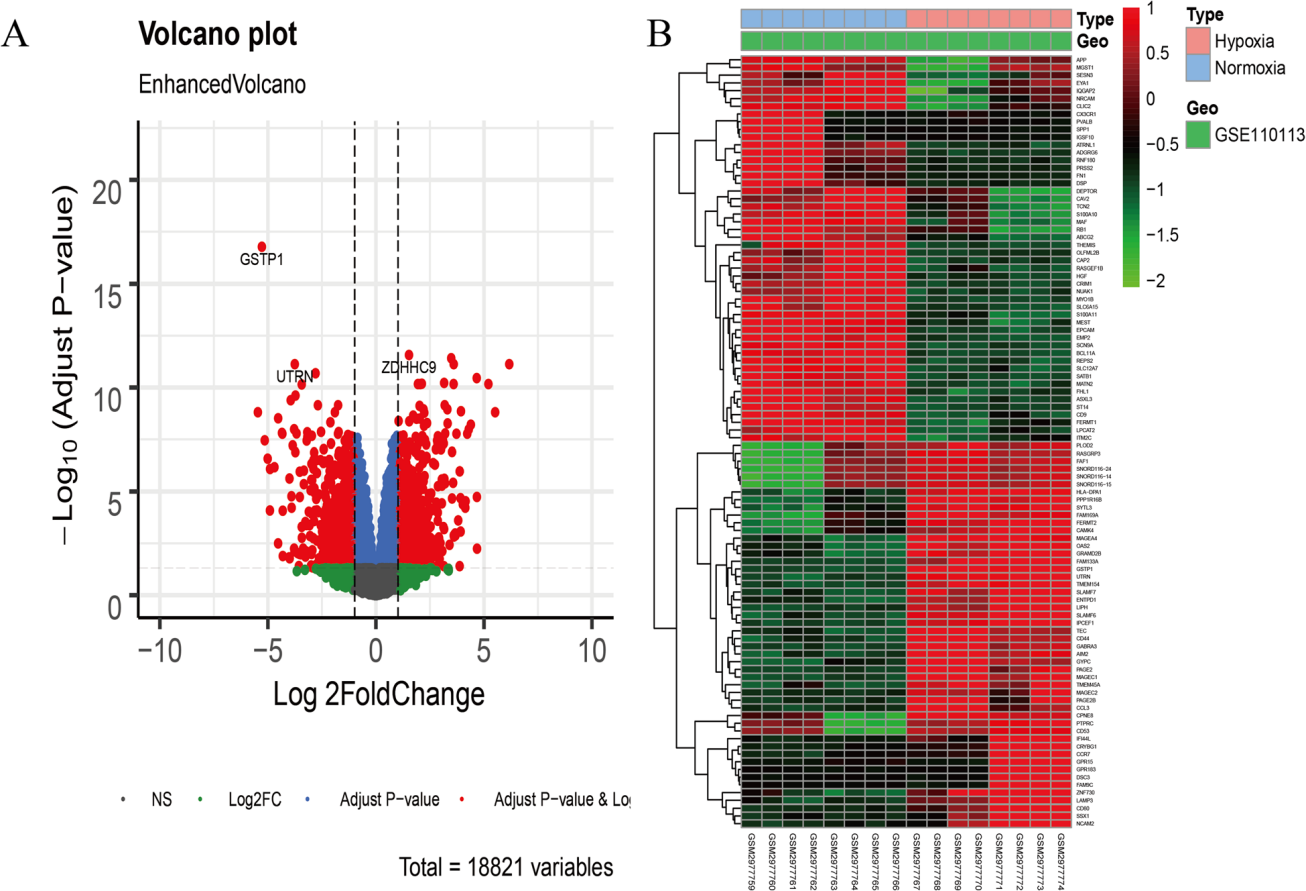
Identification of differentially expressed genes in GSE110113 dataset. **a** Volcano plot of GSE110113 dataset. Red plots represent genes with adjusted *p* value < 0.05 and [log2FoldChange (log2FC)] > 1. Other plots represent the remaining genes with no significant difference. **b** Heatmap of the top 50 DEGs (50 up- and 50 down-regulated genes). DEGs, differentially expressed genes

This study was performed as a multiple strategy to pick out the hub gene related to hypoxia in MM dataset GSE110113. The hub gene was then validated with online clinical data (Fig. 1). Myeloma cells were divided into normoxic and HR groups. Totally, 1285 DEGs were identified including 614 up-regulated and 671 down-regulated genes using “limma” R package (Fig. 2a) and a heatmap depicted top 50 genes (Fig. 2b).

### GO and KEGG enrichment analysis

**Fig. 3:**
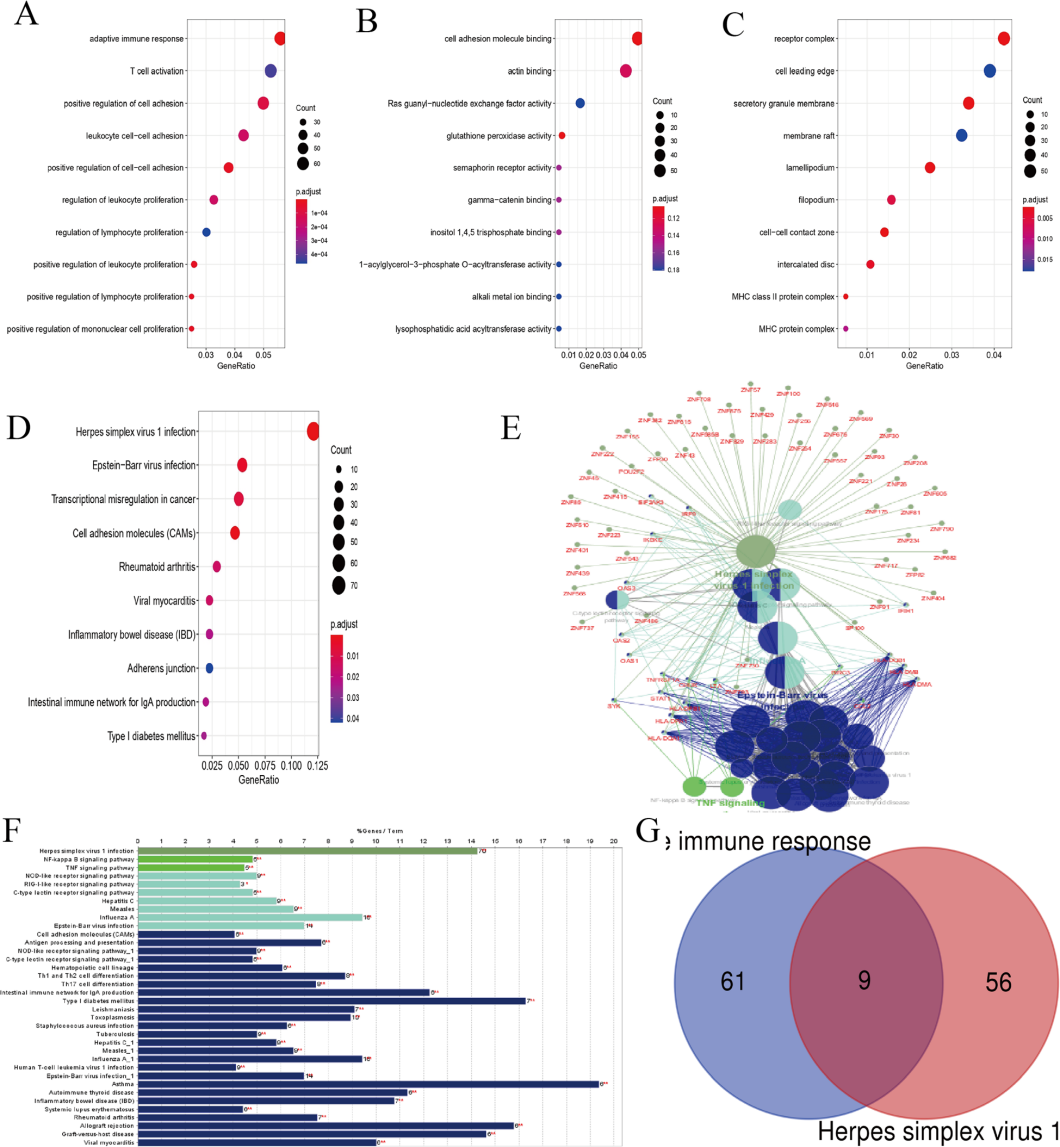
GO and KEGG enrichment analysis. **a-d** The bubble chart showed the top 10 pathways with significant difference. **a** The GO biological process enrichment analysis. **b** The GO molecular function enrichment analysis. **C** The GO cellular component enrichment analysis. **d** The KEGG enrichment analysis. **e, f** Interrelation analysis of pathways via assessment of KEGG processes in ClueGO. **e** The interrelation between pathways of KEGG. **f** Numbers of genes enriched in the identified pathways. **g** Venn diagram showed the common gene of candidate genes. GO, Gene Ontology; KEGG, Kyoto Encyclopedia of Genes and Genomes

GO and KEGG enrichment analyses were performed with all DEGs to further explore their functions with R package “clusterProfiler”. Three subontologies including BP, MF, and CC were examined in GO analysis. Adaptive immune response pathway (*p* = 1.31e-10, FDR = 6.59e-07), cell adhesion molecule binding pathway (*p* = 0.000162, FDR = 0.104) and receptor complex pathway (*p* = 1.23e-05, FDR = 0.00221) were selected as the most significant pathway in each subontologies, respectively (Fig. 3a-c). According to their *p* values, we selected adaptive immune response for further analysis and found 65 DEGs was enriched in this GO term. The top enriched pathway of the DEGs in KEGG enrichment analysis was herpes simplex virus 1 infection pathway (*p* = 1.39e-08, FDR = 3.63e-06) (Fig. 3d). We further used ClueGO to analyze and show the interrelation of the enriched pathways and the DEGs. Herpes simplex virus 1 infection pathway remained the most significant pathway, and there were 70 DEGs involved in this pathway (Figs. 3e, f).

Totally, 65 and 70 DEGs were involved in the two selected pathways, respectively (Table 1). Next, we identified 9 common genes by overlapping DEGs in the two selected pathways with Wayne diagram (Fig. 3g). They were SYK, POU2F2, LTA, HLA-DPB1, HLA-DQA1, HLA-DQB1, HLA-DPA1, HLA-DMA and HLA-DMB.

**Table 1:** DEGs identified from selected pathways of GO and KEGG

DEGs	Gene names
**Adaptive immune response pathway**	ADA, ADCY7, CD8B, DENND1B, EMP2, FAM49B, IGKV1D-8, LAIR1, PYCARD, SMAD7, SYK, THEMIS, TLR4, TNFRSF1B, TNFRSF21, ULBP3, UNC93B1, ZP3, BATF, C2, CAMK4, CD274, CD48, CD70, CD79A, CD79B, CD80, CD86, CEACAM1, CTSH, ERAP2, GPR183, HAVCR2, HLA-DMA, HLA-DMB, HLA-DPA1, HLA-DPB1, HLA- DQA1, HLA-DQB1, ICAM1, IL23A, IL23R, INPP5D, JAK3, LAMP3, LILRB4, LTA, MEF2C, NFKBIZ, PAG1, POU2F2, PTPRC, RAB27A, RORA, SAMSN1, SASH3, SLAMF1, SLAMF6, SLAMF7, SPN, TEC, TFRC, TNFAIP3, TNFSF13B, TXK
**Herpes simplex virus 1 infection pathway**	CCL2, IKBKE, SYK, TNFRSF1A, ZNF26, ZNF382, ZNF605, ZNF717, BIRC3, CHUK, EIF2AK3, HLA-DMA, HLA-DMB, HLA-DPA1, HLA-DPB1, HLA-DQA1, HLA-DQB1, IFIH1, IRF9, LTA, OAS1, OAS2, OAS3, POU2F2, SP100, STAT1, ZFP30, ZFP82, ZNF100, ZNF155, ZNF175, ZNF208, ZNF221, ZNF222, ZNF223, ZNF234, ZNF254, ZNF256, ZNF283, ZNF30, ZNF404, ZNF415, ZNF429, ZNF43, ZNF431, ZNF439, ZNF45, ZNF486, ZNF510, ZNF543, ZNF546

Abbreviations: *DEGs* differentially expressed genes; *GO* Gene Ontology; *KEGG* Kyoto Encyclopedia of Genes and Genomes

### PPI network

**Fig. 4:**
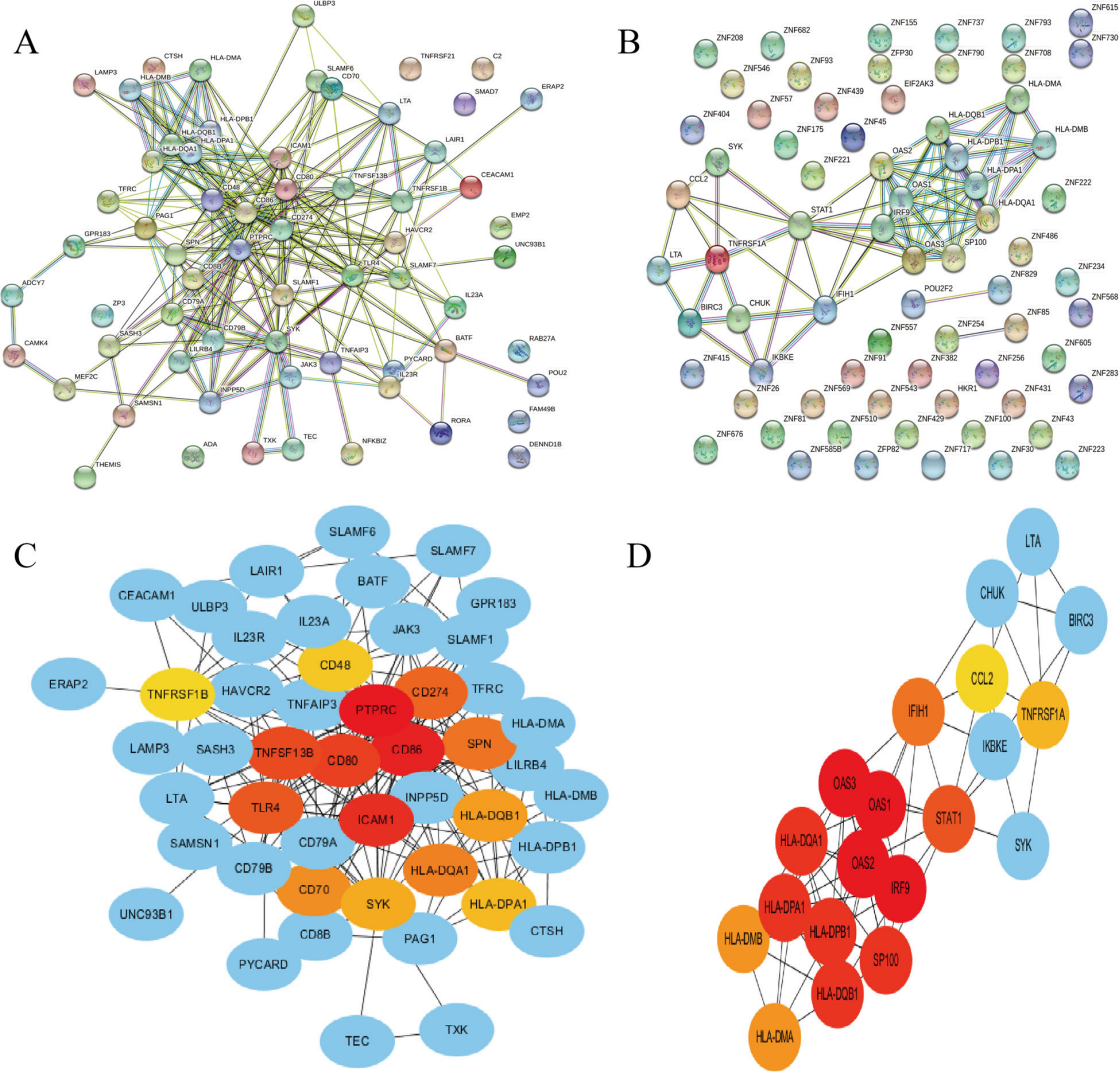
PPI network analysis. **a, b** The PPI analysis at STRING. **c, d** Cytoscape plug-ins cytoHubba analysis of candidate genes after PPI analysis. **a, c** Genes identified from adaptive immune response pathway. **b, d** Genes identified from herpes simplex virus 1 infection pathway. PPI, protein-protein interaction

To pick out and further understand the hub genes, we firstly constructed the PPI network consisting of all the DEGs from the two most significant pathways mentioned above in STRING (Fig. 4a, b), respectively. Then, we used Cytoscape plug-ins cytoHubba to screen top 15 candidate hub genes of each pathway according nodes rank (Fig. 4c, d) and they are listed in Table 2. Subsequently, we identified 3 common genes in the two sets of top 15 hub genes, including HLA-DPA1, DQHLA-DQA1 and HLA-DQB1 as candidate hub genes.

**Table 2:** The top 15 genes with the highest score of each pathway through the Cytoscape “cytoHubba” module analysis

Top 15	Adaptive immune response pathway		Herpes simplex virus 1 infection pathway	
Rank	Name	Score	Name	Score
1	PTPRC	11,394	IRF9	40,560
2	CD86	9512	OAS1	40,560
3	ICAM1	9390	OAS2	40,560
4	CD80	9146	OAS3	40,560
5	TNFSF13B	5760	SP100	40,440
6	TLR4	5337	HLA-DQB1	40,440
7	CD274	4108	HLA-DQA1	40,440
8	SPN	3648	HLA-DPB1	40,440
9	HLA-DQA1	3528	HLA-DPA1	40,440
10	CD70	2880	STAT1	250
11	HLA-DQB1	2808	IFIH1	126
12	SYK	2410	HLA-DMB	120
13	HLA-DPA1	1992	HLA-DMA	120
14	CD48	1566	TNFRSF1A	12
15	TNFRSF1B	1493	CCL2	10

### Survival analysis

**Fig. 5:**
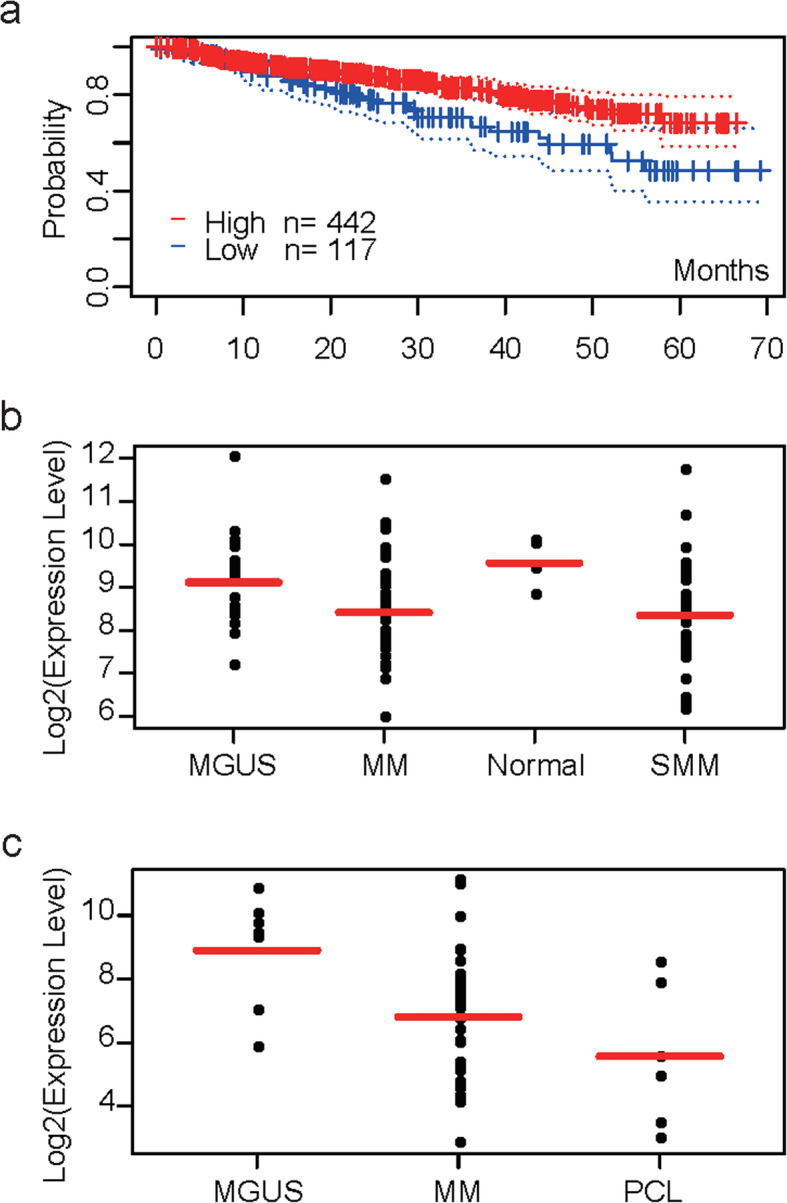
Analysis of hub gene HLA-DPA1. **a** Kaplan-Meier survival curves comparing high and low expression of HLA-DPA1 in MM with PrognoScan (Cox *p* = 0.005411). **b, c** HLA-DPA1 gene expression in different clinical datasets. **b** HLA-DPA1 gene expression in GSE47552 dataset (*p* = 0.017). **c** HLA-DPA1 gene expression in GSE2113 dataset (*p* = 0.007). MGUS, monoclonal gammopathy of undetermined significance; MM, multiple myeloma; SMM, smoldering multiple myeloma; PCL, plasma-cell leukemia

Finally, we evaluated the correlation between candidate hub genes and the prognosis of patients with MM. Potential prognostic value of the candidate hub genes were assessed with PrognoScan. The result showed that only HLA-DPA1 (Cox *p* = 0.005411) was statistically significant associated with DSS of MM patients based on 559 patients in GSE2658 dataset (Fig. 5a, Additional file 1). In addition, ScanGEO exploration results showed expression level of HLA-DPA1 in MM patients was significant lower than that in HDs (*p* = 0.017) according to GSE47552 dataset (Fig. 5b). The clinical characteristics of the MM patients [20] in GSE47552 dataset is showed in Additional file 2. Regarding GSE2113, there are 7 monoclonal gammopathy of undetermined significance (MGUS), 39 newly diagnosed MM and 6 plasma-cell leukemia (PCL) patients. As the severity of the disease woresned, the level of HLA-DPA1 gene expression gradually decreased (*p* = 0.007) (Fig. 5c). Further verification of this gene in clinical research remains need.

## Discussion

In this study, we analyzed 1285 DEGs between normoxic and hypoxic cultured MM cells based on GSE110113 dataset. Enrichment analysis indicated that adaptive immune response was the most significant GO term and herpes simplex virus 1 infection pathway was the most significant KEEG pathway. It is well-known that human immune system can eradicate cancer cells. Cancers’ occurrence and development is critically associated with immune response adaptation and immune escape which have been demonstrated with mice model [22, 23]. Herpes simplex virus (HSV) 1 has antitumor effect which mainly depends on its cytotoxic effect and replication ability with tumor in order to produce more virus for tumor lysis [24]. Previous study indicated HSV was associated with occurrence of MM and Bortezomib could inhibit HSV infection by halting viral capsid transport to the nucleus [25].

Establishment of the PPI network and further analysis with Cytoscape plug-ins cytoHubba identified 3 candidate hub genes, HLA-DPA1, HLA-DQA1 and HLA-DQB1. The major histocompatibility complex (MHC) class II proteins include HLA-DR, HLA-DQ and HLA-DP classical proteins and they only expressed on professional antigen-presenting cells (B lymphocytes, dendritic cells and macrophages) to activate CD4+ T cells [26]. They could participate in cancer development as it has been proved that dysregulation of immune function which involved antigen presentation was associated with cancer [27]. Subsequently, survival analysis based on GSE2658 dataset with PrognoScan revealed HLA-DPA1 as the hub gene associated with DSS of MM patients. Since GSE2658 dataset did not provided detail clinical data of patients’ general condition, multivariate Cox’s proportional hazard regression models could not be constructed to further clarify the relationship between HLA-DPA1 and survival. According to ScanGEO analysis results, gene expression of HLA-DPA1 was significantly lower compared to HDs and MGUS.

Hypoxia is common and essential in various cancers which can bring different gene expression change during metabolic adaptations [28]. As a result, cancer cells can survival and keep high rate proliferation. Previous studies have shown hypoxic bone marrow microenvironment plays a critical role in MM occurrence and progression through different aspects. For instance, endothelial cells (ECs) in MM patients having a hypoxic phenotype could keep up with enhanced angiogenesis in cancer growth and metastasis [8]. Hypoxia induced MM cells dedifferentiation, stem-cell like state acquisition without apoptosis and enhanced drug resistance to proteasome inhibitors [9].

In the GO enrichment analysis, cell adhesion molecule binding was the most significant term. Evidences suggested cell adhesion molecule binding is an important pathway in MM related to hypoxia. Hypoxia reduces the adhesion of tumor cells and accelerates tumor development process [7, 29], manifested as extramedullary (EMD). Central nervous system (CNS) involvement phenotype is an rare, EMD form of MM which indicates unfavorable cytogenetics, shorter survival time even with intensive treatment [30]. Capicua transcriptional repressor (CIC) is a transducer of receptor tyrosine kinase (RTK) signaling that functions through default repression [31]. Marra MA et al. found that CIC deficiency was associated with down-regulated expression of genes involving in cell-cell adhesion which led to tumor progression and over-expression mitogen-activated protein kinase (MAPK) signaling cascade [32]. Another research proved CIC mutation affected the BRAF-RAS pathway and resulted in drug resistance in MM patients [33]. Other several mutations including Ki-ras2 Kirsten rat sarcoma viral oncogene homolog (KRAS), Neuroblastoma Ras viral oncogene homolog (NRAS) also participate in drug resistance of MM [34, 35]. In our study, HLA-DPA1 is also down-regulated under hypoxic condition and we hypothesize that it may play an oncogenic role in MM through hypoxic activated signaling pathway.

HLA-DPA1, also known as HLA-DP1A, HLASB or DPA1, belongs to the HLA class II alpha chain paralogues [36]. As a result, HLA-DPA1 function as an MHC class II receptor to participate in immune response and antigenic peptides presentation. Clinical study on adrenocortical tumors (ACT) indicated low expression of HLA-DPA1 was associated with poor prognosis [37]. Acute myeloid leukemia (AML) relapse after transplantation was analyzed by Christopher MJ et al. It was proved to be associated with dysregulation of pathways which had an influence on immune function. HLA-DPA1 and several other MHC class II genes’ down-regulation were involved as they function in antigen presentation [38]. Other several researches showed MHC class II genes had crucial relationship with cancer immunology, and down-regulation of related genes indicated a poor prognosis [26, 39, 40].

## Conclusion

HLA-DPA1 was a hub gene related to hypoxia in MM. Down-regulated expression of HLA-DPA1 was associated with shorter survival time of MM patients. Notably, 3 candidate hub genes were all related to immune response. Based on the findings in our study, further researches investigating immune process of MM pathogenesis may help us to better understand MM. This study provided a novel insight into HLA-DPA1 as a critical potential biomarker for MM.

## Supplementary information

**Additional file 1.**

**Additional file 2.**

## Data Availability

The dataset analysed during the current study are available in the NCBI-GEO repository, https://www.ncbi.nlm.nih.gov/geo/query/acc.cgi?acc=GSE110113.

## Abbreviations

MM: Multiple myeloma
NCBI-GEO: National Center for Biotechnology Information-Gene Expression Omnibus
HLA-DPA1: Major histocompatibility complex, class II, DP alpha 1
DEGs: Differentially expressed genes
GO: Gene Ontology
KEGG: Kyoto Encyclopedia of Genes and Genomes
PPI: Protein-protein interaction
MCC: Maximal Clique Centrality
HR: Hazard ratio
DSS: Disease specific survival
HDs: Healthy donors
ASCT: Autologous stem cell transplant
PFS: Progression-free survival
OS: Overall survival
HR: Hypoxia-resistant
log2FC: log2FoldChange
BP: Biological process
MF: Molecular function
CC: Cellular component
MGUS: Monoclonal gammopathy of undetermined significance
PCL: Plasma-cell leukemia
HSV: Herpes simplex virus
MHC: Major histocompatibility complex
ECs: Endothelial cells
EMD: Extramedullary
CNS: Central nervous system
CIC: Capicua transcriptional repressor
RTK: Receptor tyrosine kinase
MAPK: Mitogen-activated protein kinase
KRAS: Ki-ras2 Kirsten rat sarcoma viral oncogene homolog
NRAS: Neuroblastoma Ras viral oncogene homolog
ACT: Adrenocortical tumors
AML: Acute myeloid leukemia
