# Supplementary figures and images for: HLA-DPA1 gene is a potential predictor with prognostic values in multiple myeloma
Source: BMC Cancer. 2020 Sep 24;20:915. doi: 10.1186/s12885-020-07393-0 (PMC7513295; doi:10.1186/s12885-020-07393-0)

HLA-DQA1

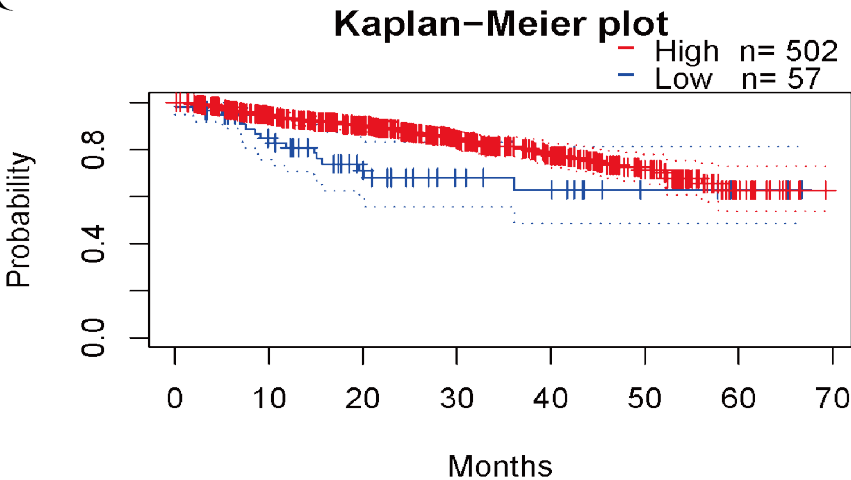

COX p=0.126574  
HR [95% CI]=0.88 [0.76-1.04]

HLA-DQB1

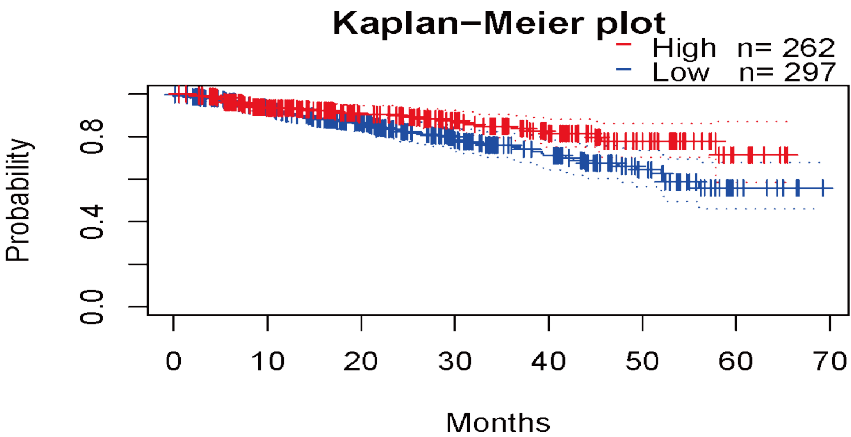

COX p=0.079639  
HR [95% CI]=0.85 [0.71-1.02]

Supplement: Supplementary file 1 — Additional file 1. [file 12885_2020_7393_MOESM1_ESM.pdf]
