# Supplementary material for: HLA-DPA1 gene is a potential predictor with prognostic values in multiple myeloma
Source: BMC Cancer. 2020 Sep 24;20:915. doi: 10.1186/s12885-020-07393-0 (PMC7513295; doi:10.1186/s12885-020-07393-0)
Supplement: Supplementary file 2 — Additional file 2. [file 12885_2020_7393_MOESM2_ESM.docx]

**Additional file 2: Clinical characteristics of patients**

| **Characteristics** | **MGUS** | **SMM** | **MM** |
| --- | --- | --- | --- |
| **Sex (%)** |  |  |  |
| Female | 32 | 48 | 53 |
| Male | 68 | 52 | 47 |
| **Age (years)** |  |  |  |
| Median (range) | 78(39-85) | 65(39-84) | 67(41-81) |
| **Plasma cells (%)** |  |  |  |
| Median (range) | 4(2-8) | 14(4-48) | 37(10-95) |
| **Ig subtype (%)** |  |  |  |
| IgA | 68 | 55 | 58 |
| IgG | 32 | 45 | 34 |
| Bence-Jones protein | 0 | 0 | 8 |

Abbreviations: MGUS, monoclonal gammopathy of undetermined significance; SMM, smoldering multiple myeloma; MM, multiple myeloma.
